# Application of ^1^H-NMR combined with qRT-PCR technology in the exploration of rosmarinic acid biosynthesis in hair roots of *Salvia miltiorrhiza* Bunge and *Salvia castanea* f*. tomentosa* Stib

**DOI:** 10.1007/s00425-020-03506-y

**Published:** 2020-11-27

**Authors:** Zhuoni Hou, Yuanyuan Li, Feng Su, Jipeng Chen, Xiaodan Zhang, Ling Xu, Dongfeng Yang, Zongsuo Liang

**Affiliations:** 1 The Key Laboratory of Plant Secondary Metabolism and Regulation of Zhejiang Province, College of Life Sciences and Medicine, Zhejiang Sci-Tech University, Hangzhou, 310018 China; 2 Key Laboratory for Green Pharmaceutical Technologies and Related Equipment of Ministry of Education, College of Pharmaceutical Sciences, Zhejiang University of Technology, 18 Chao Wang Road, Hangzhou, 310014 China

**Keywords:** ^1^H-NMR, Methyl jasmonate, qRT-PCR, Rosmarinic acid, *Salvia castanea Diels* f. *tomentosa* Stib, *Salvia miltiorrhiza* Bunge

## Abstract

**Main conclusion:**

Methyl jasmonate promotes the synthesis of rosmarinic acid in *Salvia miltiorrhiza* Bunge and *Salvia castanea* f. *tomentos*a Stib, and it promotes the latter more strongly.

**Abstract:**

*Salvia miltiorrhiza* Bunge (SMB) is a traditional Chinese medicinal material, its water-soluble phenolic acid component rosmarinic acid has very important medicinal value. *Salvia castanea* f*. tomentosa* Stib (SCT) mainly distributed in Nyingchi, Tibet. Its pharmacological effects are similar to SMB, but its rosmarinic acid is significantly higher than the former. Methyl jasmonate (MJ) as an inducer can induce the synthesis of phenolic acids in SMB and SCT. However, the role of MJ on rosmarinic acid in SMB is controversial. Therefore, this study used SMB and SCT hair root as an experimental material and MJ as a variable. On one hand, exploring the controversial reports in SMB; on the other hand, comparing the differences in the mechanism of action of MJ on the phenolic acids in SMB and SCT. The content of related metabolites and the expression of key genes in the synthesis pathway of rosmarinic acid was analyzed by ^1^H-NMR combined with qRT-PCR technology. Our research has reached the following conclusions: first of all, MJ promotes the accumulation of rosmarinic acid and related phenolic acids in the metabolic pathways of SMB and SCT. After MJ treatment, the content of related components and gene expression are increased. Second, compared to SMB, SCT has a stronger response to MJ. It is speculated that the different responses of secondary metabolism-related genes to MJ may lead to different metabolic responses of salvianolic acid between the two.

**Electronic supplementary material:**

The online version of this article (10.1007/s00425-020-03506-y) contains supplementary material, which is available to authorized users.

## Introduction

*Salvia miltiorrhiza* Bunge (SMB, Danshen in Chinese), belonging to Labiate family (Kai et al. 2011; Shi et al. 2014), is an important traditional Chinese herbal plant with long history for medicine as well as healthy food (Shi et al. 2018). It has been used clinically to treat cardiovascular diseases (Wang et al. 2017a, b, 2018; Chen and Chen 2017; Fang et al. 2018) and displays anti-inflammation (Gao et al. 2018; Wu et al. 2018), neuro-protection (Di et al. 2018; Saroya and Singh 2018), cardio-protection (Hu et al. 2012; Weng et al. 2013) and anticancer activities (Wang et al. 2017a, b; Uto et al. 2018; Qiu et al. 2018; Hsieh et al. 2018). There are two major types of pharmacologically active compounds present in SMB, one is water-soluble phenolic acids (Ma et al. 2013), and the other is fat-soluble ketones (Kim et al. 2008). *Salvia castanea Diels* f. *tomentosa* Stib (SCT), formerly known as salvia villi, also known as "Nyingchi Salvia", mainly distributed in Nyingchi, Tibet. The effective ingredients of SCT roots are similar to SMB roots, and have long been used as a substitute for SMB to treat diseases in the local area (Ye et al. 2004; Sun et al. 2004). Compared with SMB, the water-soluble component rosmarinic acid is higher in SCT, but the salvianolic acid B is lower (Yang et al. 2009). Therefore, SCT has important significance as a medicine source of rosmarinic acid.

**Fig. 1:**
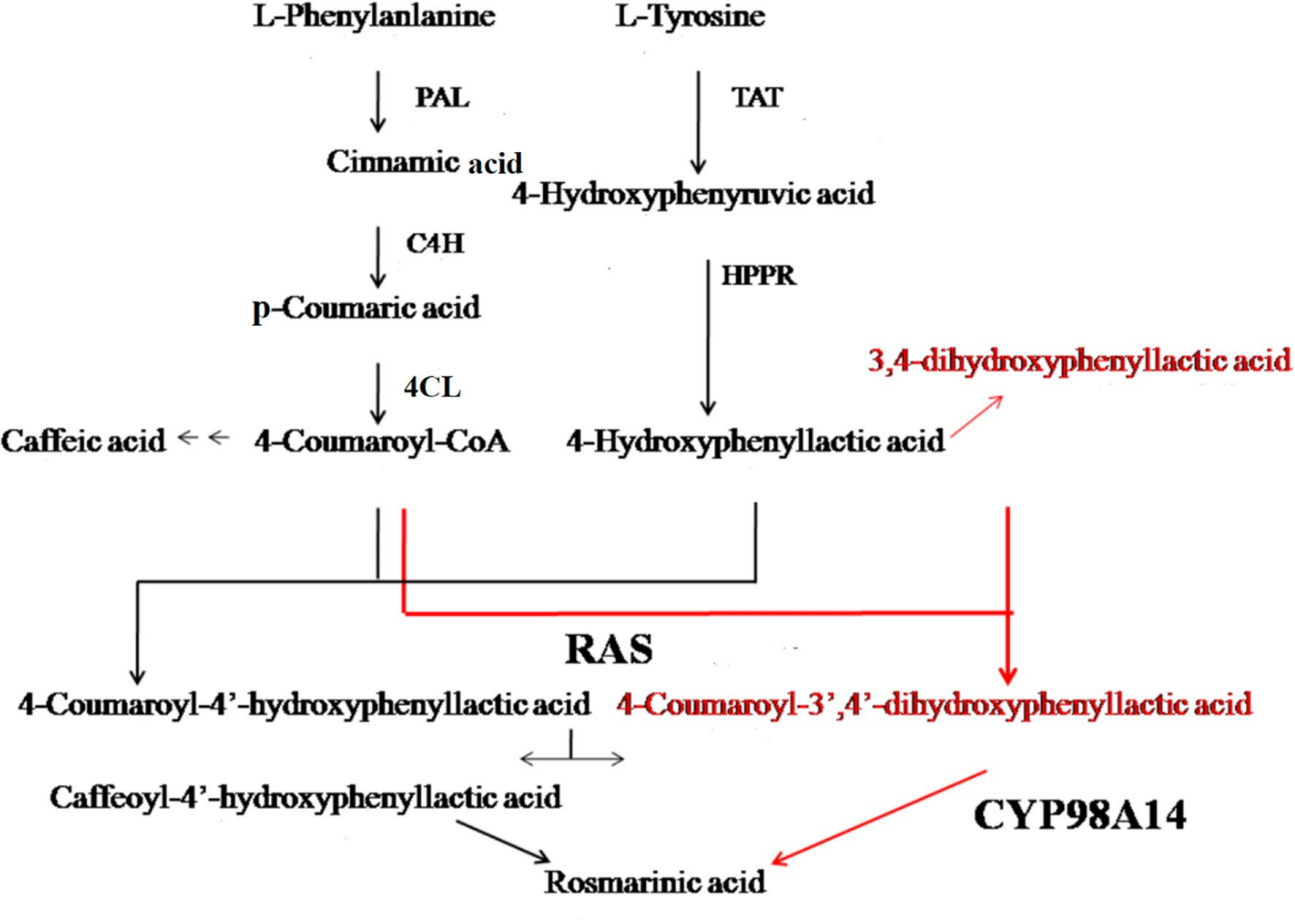
Modification of metabolic flux of rosmarinic acid in SMB

Some scholars have studied the biosynthetic pathways of phenolic acid components in SMB, and it is now generally believed that rosmarinic acid is the precursor of other more complex phenolic acid compounds. Ellis and Tower (1970) first proposed the possible source of rosmarinic acid in 1970. They fed *Mentha haplocalyx* Briq. with ^14^C radioisotope and speculated about the biogenic pathway of rosmarinic acid by separating different compounds from the young shoots. As a result, it is believed that phenylalanine and tyrosine are precursors of rosmarinic acid biosynthesis. Through the suspension culture of *Plectranthus scutellarioides* (L.) R.Br., Petersen and Simmonds (2003) comprehensively described the biogenic pathways of rosmarinic acid and related enzymes for the first time. They found that the biogenic pathway of rosmarinic acid is composed of two parallel branches, the phenylalanine branch and the tyrosine branch (Fig. S1). Afterwards, Di et al. (2013) fed the hairy root of SMB with phenylalanine labeled with ^13^C radioisotope, and corrected the metabolic flux of rosmarinic acid by liquid-mass spectrometry data analyze of different phenolic compounds in the hairy root (Fig. 1).

Methyl jasmonate (MJ) is a cyclopentanone derivative formed by the methylation of the chemical substance of jasmonic acid. It is named, because it is an important component of the flower volatiles of jasmine plants (Yan et al. 2015). It can regulate the growth and development of plants, cause cells to respond to stress, induce plants to express defense genes, synthesize defense substances, and form defense organs. It can also induce plants to produce alkaloids, terpenes, and phenols for human use (Zhu et al. 2013). MJ can induce plants to produce secondary metabolites: studying the effect of spraying MJ on pine acid, it was found that the content of coumaric acid, salicylic acid, ferulic acid, cinnamic acid and phenylacetic acid in the cones of the treatment group was higher than the control group (Wang and Yan 2012); spraying the leaves of tobacco plants with MJ can increase the nicotine content by two times (Dam et al. 2000). In addition, the effect of MJ on the active ingredient content and enzyme activity in SMB hairy root was studied (Xing et al. 2013; Xiong et al. 2018). However, the effect of MJ on rosmarinic acid is controversial. Studies have shown that MJ can significantly promote the synthesis of rosmarinic acid in SCT, but has almost no effect on SMB (Fang et al. 2017). Opposite studies have shown that MJ can promote the accumulation of rosmarinic acid (Li et al. 2013): Some evidences show that the accumulation of rosmarinic acid reaches the maximum after 24 h of MJ treatment (Li et al. 2012), and there are also studies prove that the accumulation of rosmarinic acid was the largest after treatment for 48 h by MJ (Xing et al. 2013).

Hairy root is a new technology that combines genetic engineering and cell engineering developed in the 1980s. It transforms the T-DNA contained in the Ri plasmid of agrobacterium rhizogenes into the DNA of plant cells (Yan et al. 2006), induce hairy root formation. Hairy roots are important materials for the research of root medicinal plants (Liu et al. 2015) and have important prospects in large-scale production (Wang et al. 2020). Hairy roots have a complete metabolic pathway in plant roots due to their convenient cultivation. It is an effective method for studying root medicinal materials and an effective way for large-scale production of active ingredients of Chinese medicine (Sun et al. 2014).

At present, NMR-based metabolomics and transcriptomics technologies to explore the biosynthesis of SMB phenolic acid have been applied (Liu et al. 2019). Based on the above background, this study has two purposes, one is to explore the above-mentioned controversial views, and the other is to compare the differences in the mechanism of action of MJ on SMB and SCT. We adopted ^1^H-NMR combined with qRT-PCR technology to study MJ treatment of SMB and SCT hairy roots, and the control group was treated with absolute ethanol. The content of cinnamic acid, caffeic acid, *p*-coumaric acid, l-tyrosine, l-phenylalanine, 4-hydroxyphenylpyruvic acid and rosmarinic acid (Fig. S2) were measured on 0, 1, 2, 3, 6, 9 days after treatment with MJ, combined with the expression level of key genes *PAL, TAT, 4CL, C4H, RAS, HPPR, CYP98A14* in their metabolic pathways.

## Materials and methods

### Plant materials

Hairy root of *Salvia miltiorrhiza* Bunge and *Salvia castanea* f*. tomentosa* Stib were obtained from an earlier experiment in our laboratory. Both plants were obtained from Tasly Pharmaceutical Group Co. LTD (Tianjin, China). We transferred the cultures from solid medium to hormone-free MS liquid medium with 30 g L^−1^ sucrose and cultivated them at 25 °C in the dark. The induction experiment was conducted after stable growth reached. Specifically, induction was started 18 days after 3 g of hairy roots was inoculated into 250-mL Erlenmeyer flasks through the application of the abiotic elicitor MJ (0.1 mM) as previously described (Xiao et al. 2009). Hairy roots were harvested at 0, 1, 2, 3, 6 and 9 days post-induction.

## Methods

### MJ dosage and solution preparation

MJ is made up of 50% mother liquor with absolute ethanol as a co-solvent. Sterilize through a 0.22 μm microporous membrane and add to the medium to make the final MJ concentration 200 μmol L^−1^ (Wang et al. 2007). The control group added equal amount of absolute ethanol.

### Sample preparation

SMB and SCT hairy roots with different induction time removed from the culture medium. The culture medium was washed away with tap water and blotted with absorbent paper. Take a part of the 40 °C oven to dry to constant weight, cool at room temperature, the hairy root after drying is used as the material for determining the content of phenolic acid active ingredients. Another part was wrapped in tin foil paper and then liquid nitrogen was cooled instantly and frozen in a − 80 °C refrigerator, as a material for measuring the expression of key genes.

### ^1^H-NMR determination of phenolic acids in hairy root of SMB

Grind the dried hairy roots of SMB and SCT to powder, add 70% methanol solution to soak overnight, then ultrasonically extract for 45 min, centrifuge at 3214*g* for 15 min, filter liquor was transferred into a clean Eppendorf tube and evaporated to dryness under nitrogen at 35 °C, then was added 600 μL D_2_O (contain proper amount of TMSP) to dissolve for NMR analysis. The NMR sample tube with sample was assembled for analysis at 298 K and sealed and prior to ^1^H-NMR measurement. Samples for NMR analysis were measured on a 600 MHz AVANCE III HD spectrometer with a TXI probe at 298 K. The solvent-suppression was used in the acquisition of NMR spectra. ^1^H-NMR experimental parameters were shown as follows: Lc1pnf2 pulse sequence (1D version of noesygppr using presaturation during relaxation delay and mixing time) with 32 scans of 32 K data points in a spectral width of 12,626.26 (21 ppm), acquisition time 5.0 s, relaxation delay 25 s (depending on the longest longitudinal relaxation time referring to the IS determined by the Bruker inversion recovery pulse program). All the data processing was performed by using MestReNova 11.0.

### Internal standards

To test internal standards, the extracted sample M9 with 600 μL D_2_O, containing appropriate amount of TMSP, maleic acid and 3,4,5-trichloropyridine as IS were analyzed.

### Quantitative NMR analysis

The most important fundamental relation of qNMR is signal response (integrated signal area) *I*_*x*_ in a spectrum that is directly proportional to the number of nuclei *N*_*x*_ generating the corresponding resonance line: (Malz and Jancke 2005; Gadape and Parikh 2011; Li et al. 2020):

1 $$ I_{x} = K_{{\text{s}}} N_{x} , $$

*K*_s_ is an unknown spectrometer constant, which is a constant for all resonance lines in the same ^1^H single-pulse NMR spectrum. Accordingly, the determination of relative area ratios *I*_*x*_/*I*_*y*_ is the most efficient way to obtain quantitative results using Eq. (2) when *K*_s_ cancels for the ratio:

2 $$ \frac{{I_{x} }}{{I_{y} }} = \frac{{N_{x} }}{{N_{y} }}. $$

For the purity determination of a substance an internal standard with known purity is needed. Based on Eq. (2), the component purity can be calculated from the NMR intensity via the following equations:

3 $$ W_{x} = \frac{{I_{x} \times N_{{{\text{Std}}}} \times M_{x} \times m_{{{\text{Std}}}} }}{{I_{{{\text{Std}}}} \times N_{x} \times M_{{{\text{Std}}}} }} $$

4 $$ P_{X} = \frac{{I_{X} \times N_{{{\text{Std}}}} \times M_{X} \times m_{{{\text{Std}}}} \times P_{{{\text{Std}}}}}}{{I_{{{\text{Std}}}} \times N_{X} \times M_{{{\text{Std}}}} \times m}}, $$

*W*_*x*_ and *P*_*x*_ represent the mass and purity of the analyte. *M*_*x*_ and *M*_Std_ are the molar masses of the analyte and the standard (TMSP 172.17 g mol^−1^). *m* is the weighed mass of the investigated sample. *m*_Std_ and *P*_Std_ are the weighed mass and the purity (99.5%) of the standard. *N*_Std_ and *I*_Std_ correspond to the number of protons for the standard (in this experiment is 9) and the integrated signal area of a typical NMR line (which was 9 in this experiment). *N*_*x*_ and *I*_*x*_ correspond to the number of protons for the analyte ^1^H.

### qRT-PCR to measure gene expression of key genes

Total RNA for qRT-PCR analysis was extracted from frozen samples using polysaccharide polyphenol plant total RNA extraction kit (Tiangen Biochemical Technology Co., Ltd.), following the manufacturer’s protocol. The yield and integrity of RNA were assessed using a NanoDrop Micro Photometer (Thermo Scientific, USA) and agarose gel electrophoresis, respectively. cDNA was synthesized from 500 ng of total RNA using a cDNA synthesis kit (Takara), following the manufacturer’s protocol. Specific primers for seven key genes to be detected were designed (Table 1) and synthesized by Zhejiang Youkang Biotechnology Co., Ltd. The internal reference gene used housekeeping gene 18S rRNA to design primers F18S (ATGATAACTCGACGGATCGC) and R18S (CTTGGATGTGGTAGCCGTTT), and the same template was used for qRT-PCR amplification of the participating samples.

**Table 1:** Primers for real-time quantitative PCR

Genes	Forward primer (5′ → 3′)	Reverse primer (5′ → 3′)	Genbank ID
PAL1	GGCGGCGATTGAGAGCAGGA	ATCAGCAGATAGGAAGAGGAGCACC	EF462460.1
TAT1	TTCAACGGCTACGCTCCAACT	AAACGGACAATGCTATCTCAAT	DQ334606.1
C4H1	CCAGGAGTCCAAATAACAGAGCC	GAGCCACCAAGCGTTCACCAA	DQ355979.1
4CL2	TCGCCAAATACGACCTTTCC	TGCTTCAGTCATCCCATACCC	AY237164.1
HPPR1	GACTCCAGAAACAACCCACATT	CCCAGACGACCCTCCACAAGA	DQ099741.1
RAS	CGCCCTAGTTGAGTTCTACCCTTACGC	TCGGATAGGTGGTGCTCGTTTGC	FJ906696.1
CYP98A14	CTAAGGAGGTGCTGAAGGAG	GTGGAGTCGTTGTAGATGGA	HQ316179.1

PCR reactions (10 μL) included 1.5 μL cDNA, 0.4 μL of each primer, 5 μL SYBR Premix, 0.1 μL Rox Reference Dye II and 2.6 μL H_2_O. Reactions were performed using an ABI Quant Studio 6 Flex real-time PCR system (Applied Biosystems, USA) under the following conditions (Xing 2015): 95 °C for 30 s, 40 cycles of 95 °C for 5 s, and 60 °C for 30 s, followed by 95 °C for 15 s, 60 °C for 1 min, and 95 °C for 15 s to obtain melt curves. The expression of each gene relative to average *C*_t_ values of the housekeeping genes was determined and analyzed using ABI 6 Flex System Sequence Detection Software (Applied Biosystems, USA). Quantification of the relative changes in gene transcript level was performed in accordance to the 2 − ^ΔΔ*C*t^ method (Livak and Schmittgen 2001). For control samples (this refers to samples processed by MJ for 0 day), the mean relative expression level of the assayed gene was assigned a value of 1.0, and the relative expression level of all samples calculated relative to it. Results represent the mean of three biological replicates.

## Results

### Identification of metabolites in MJ-induced SMB hairy root cultures through ^1^H-NMR

#### Specificity and selectivity

**Fig. 2:**
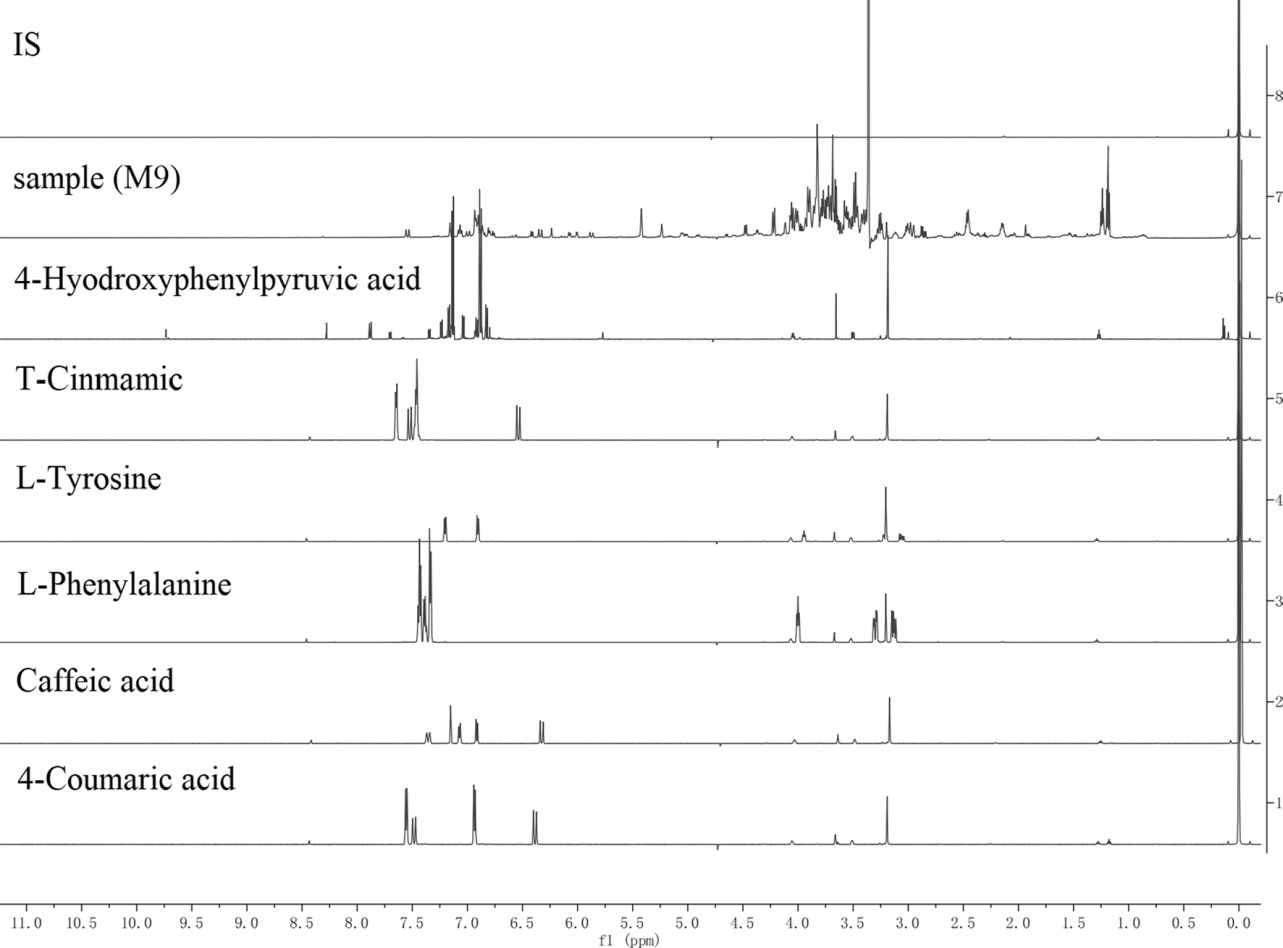
^1^H-NMR spectra of IS, sample (M9), 4-hyodroxyphenylpyruvic acid, cinmamic acid, l-tyrosine, l-phenylalanine, caffeic acid and *p*-coumaric acid

**Fig. 3:**
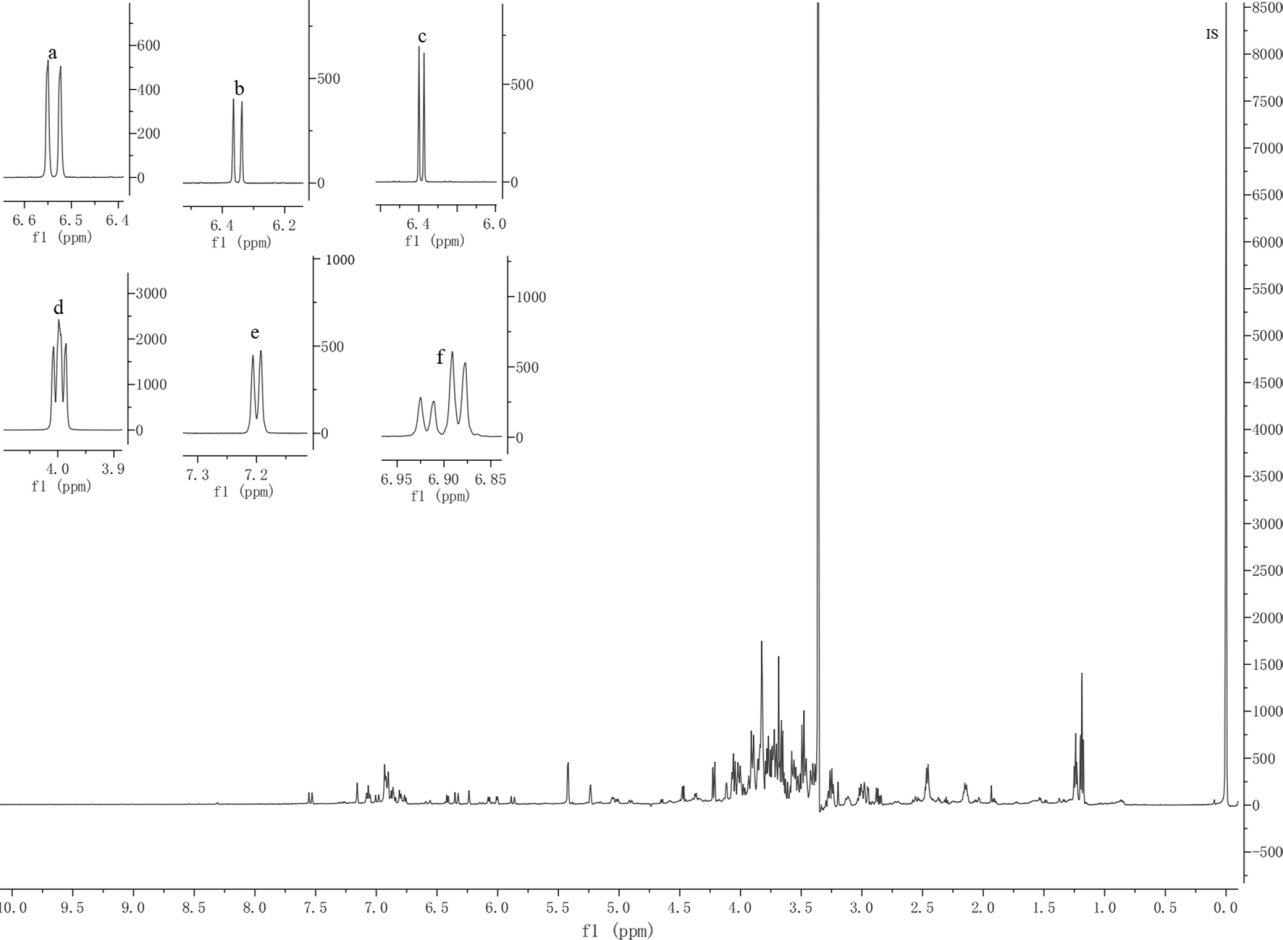
Representative ^1^H-NMR spectra for cinnamic acid (**a**), caffeic acid (**b**), *p*-coumaric acid (**c**), l-phenylalanine (**d**), l-tyrosine (**e**) and 4-hydroxyphenylpyruvic acid (**f**) with IS in the full range of 0–10.0 ppm

The sample solutions were optimized to obtain the best separation and stability for all the integrated signals in ^1^H-NMR spectrogram. Quantification was performed by an NMR sample-tube adapter at 298 K. The result was found to give the desired signal separation of *p*-coumaric acid, l-phenylalanine, cinnamic acid, caffeic acid, l-tyrosine and 4-hydroxyphenylpyruvic acid (Fig. 2). As a necessary prelude to all the measurements, analyte and IS were analyzed qualitatively by routine ^1^H experiments to determine longest spin–lattice relaxation time, usually at least five times the T1, thus the optimized relaxation delay of 25 s was obtained. Based on the optimized NMR parameters, signals for cinnamic acid at 6.54 ppm, caffeic acid at 6.34 ppm, *p*-coumaric acid at 6.40 ppm, l-phenylalanine at 3.99 ppm, l-tyrosine at 7.18 ppm and 4-hydroxyphenylpyruvic acid at 6.88 ppm were selected as the quantification signals (Fig. 3). The TMSP was chosen as the IS due to its good solubility and stability.

### Linearity, LOD and LOQ

The intensity of the response signal is directly proportional to the amount of nuclei, consequently, the linearity regression yielded a good correlation coefficient (*r*^2^ > 0.985). The concentration ratios of the six references ranged from 0.01 to 1.00 mg mL^−1^ (cinnamic acid; caffeic acid; l-tyrosine), 0.01 to 1.40 mg mL^−1^ (*p*-coumaric acid), 0.02 to 1.60 mg mL^−1^ (l-phenylalanine) and 0.01 to 1.20 mg mL^−1^ (4-hydroxyphenylpyruvic acid), respectively. The limit of detection (LOD) presents the lowest detectable analyte concentration, whilst the limit of quantitation (LOQ) represents the lowest quantifiable analyte concentration. These are two fundamental elements of method validation defining the limitations of an analytical method. In qNMR, the LOD and LOQ cannot be determined by means of SNR (signal noise ratio) as the NMR response signals are Lorentzian lines. Hence, the LOD and LOQ were determined using the standard deviation of the response *σ* and the slope *S* of a calibration curve obtained in the linearity study by the following equations:

5 $$ {\text{LOD}} = \frac{3.3\sigma }{S} $$

6 $$ {\text{LOQ}} = \frac{10\sigma }{S}. $$

The result of linearity, LOD and LOQ are shown in Table 2.

**Table 2:** Linearity, LOD and LOQ of six phenolic acids

References	Calibration curves	Range (mg mL^−1^)	*r*^2^	LOD (mg mL^−1^)	LOQ (mg mL^−1^)
Cinmamic acid	*Y* = 1.152*X* + 0.001	0.01–1.00	1.000	1.81	5.48
Caffeic acid	*Y* = 1.447*X* − 0.001	0.01–1.00	1.000	2.63	7.98
*p*-Coumaric acid	*Y* = 1.589*X* − 0.012	0.01–1.40	0.999	0.65	1.97
l-Phenylalanine	*Y* = 1.034*X* + 0.002	0.02–1.60	0.997	0.57	1.74
l-Tyrosine	*Y* = 1.156*X* − 0.009	0.01–1.00	0.999	0.38	1.16
4-Hydroxyphenylpyruvic acid	*Y* = 0.930*X* + 0.030	0.01–1.20	0.987	0.34	1.02

### Real sample determination

**Fig. 4:**
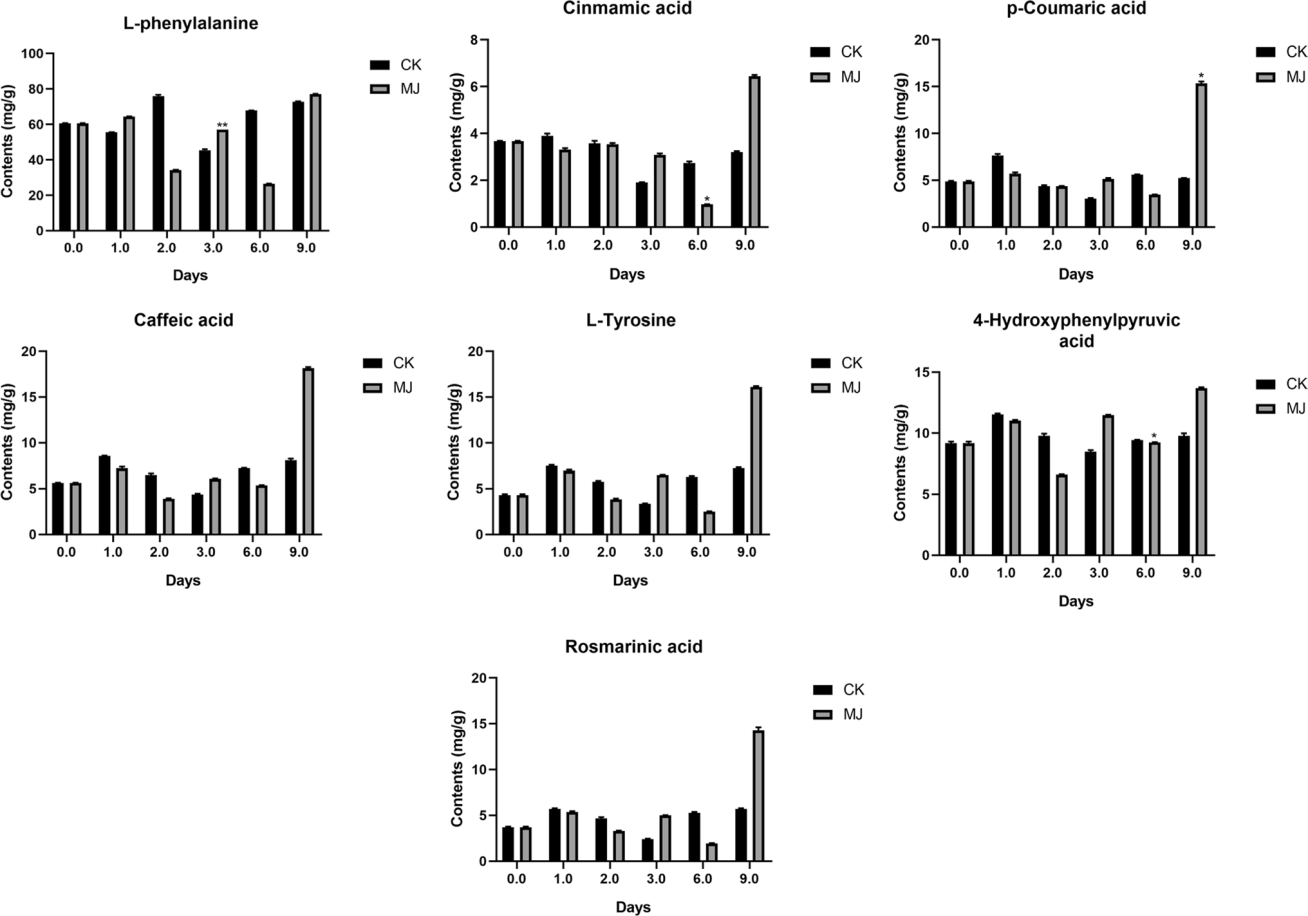
Contents of phenolic acids in SMB under different treatments by ^1^H-NMR. *In the figure indicates that the treatment group data and the control group at the same harvest time point are significantly different at the *p* < 0.05 level. The data in the figure is the average of three replicates, and the error bar shows the standard deviation

**Fig. 5:**
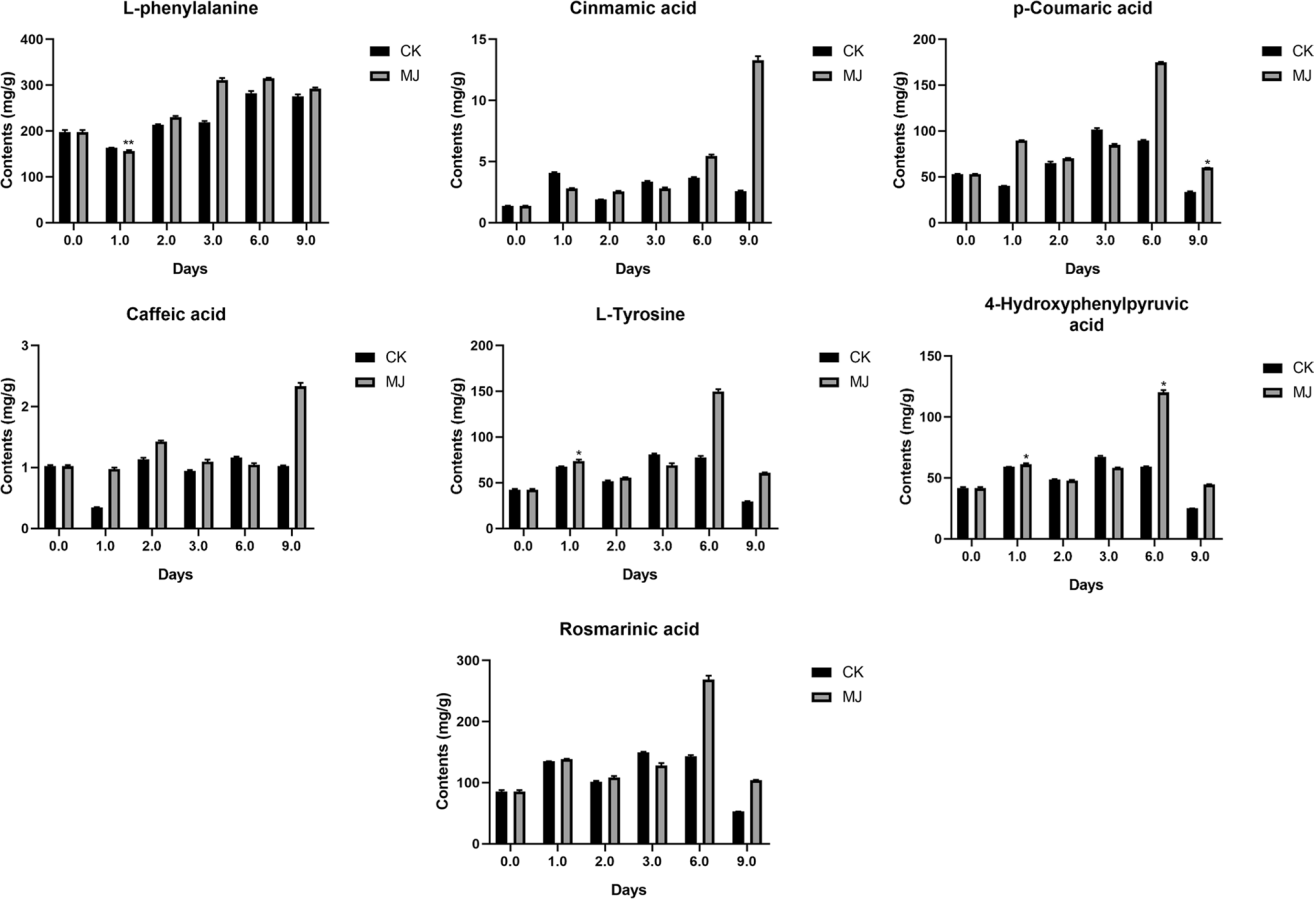
Contents of phenolic acids in SCT under different treatments by ^1^H-NMR. *In the figure indicates that the treatment group data and the control group at the same harvest time point are significantly different at the *p* < 0.05 level. The data in the figure is the average of three replicates, and the error bar shows the standard deviation

We used ^1^H-NMR to quantitatively analyze six phenolic acids in SMB (Fig. 4) and SCT (Fig. 5), and calculated the content of rosmarinic acid (Hou et al. 2020).

As the picture shown, the contents of l-phenylalanine, *p*-coumaric acid, l-tyrosine and 4-hydroxyphenylpyruvic acid in SCT were higher than that in SMB. Before MJ treatment, the content of cinnamic acid was not much different in the both, but after MJ treatment, the content of cinnamic acid in SCT was higher than that in SMB. Especially when MJ was treated for 9 days, the content of the former was about twice that of the latter. Interestingly, after 6 days of MJ treatment, the cinnamic acid content in the SMB treatment group was significantly lower than the control group. No matter before and after MJ treatment, the content of caffeic acid in SMB was higher than that in SCT. It is speculated that this is related to the by-pass status of caffeic acid in the synthesis of rosmarinic acid, which also explains the difference in the content of rosmarinic acid between the two. After 9 days of MJ treatment, the content of *p*-coumaric acid in SMB and SCT and 4-hydroxyphenylpyruvic acid in SCT were significantly higher than that in the control group. In addition, when MJ was treated for 1 day, the content of l-tyrosine and 4-hydroxyphenylpyruvic acid in SCT was also significantly higher than that in the control group.

### Gene expression was analyzed by qRT-PCR

**Fig. 6:**
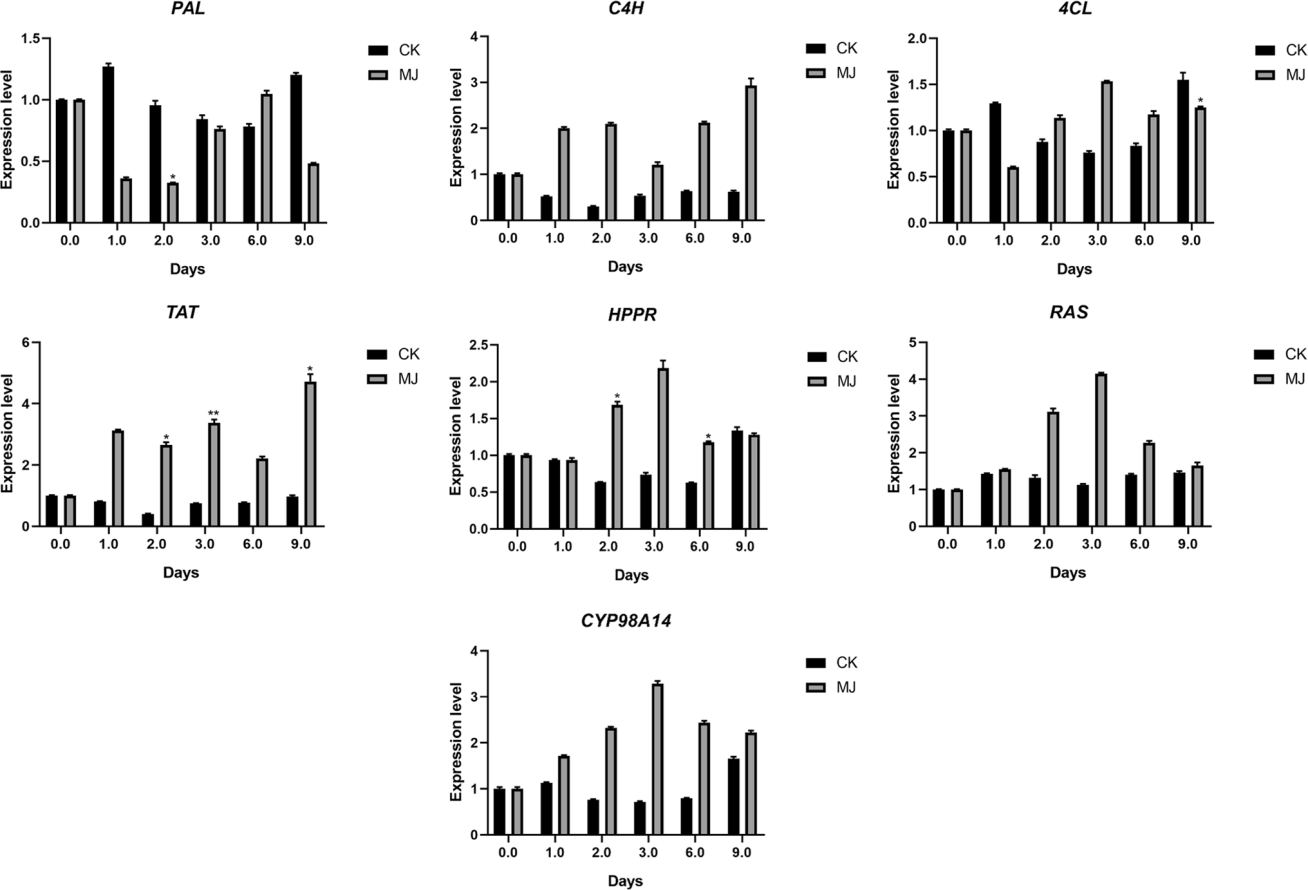
qRT-PCR determination of related gene expression in phenolic acids metabolism pathway under different treatments of SMB. *In the figure indicates that the treatment group data and the control group at the same harvest time point are significantly different at the *p* < 0.05 level. The data in the figure is the average of three replicates, and the error bar shows the standard deviation

**Fig. 7:**
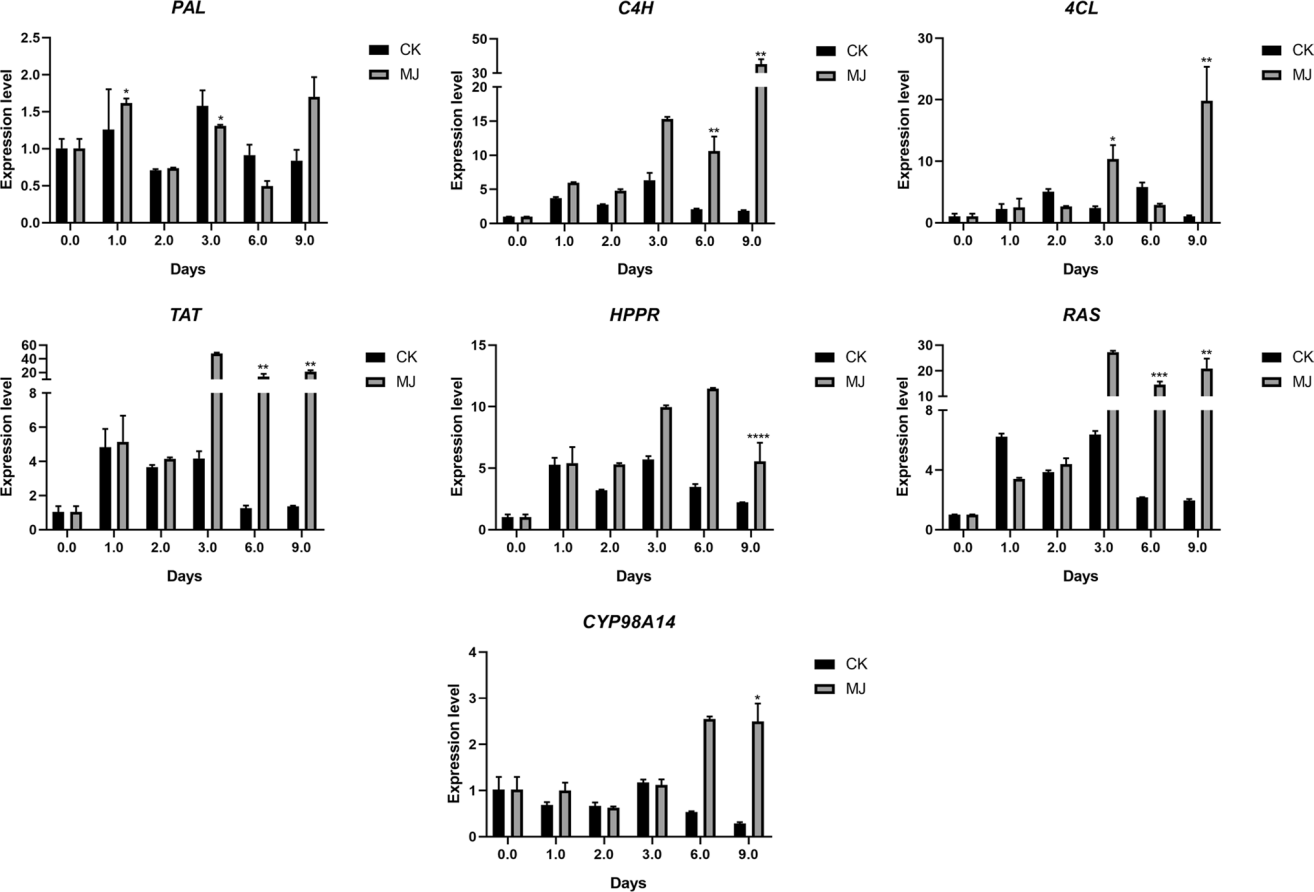
qRT-PCR determination of related gene expression in phenolic acids metabolism pathway under different treatments of SCT. *In the figure indicates that the treatment group data and the control group at the same harvest time point are significantly different at the *p* < 0.05 level. The data in the figure are the average of three replicates, and the error bar shows the standard deviation

qRT-PCR was performed to investigate the expression levels of seven key genes in the rosmarinic acid synthesis pathway in the hairy roots of SMB (Fig. 6) and SCT (Fig. 7) treated with MJ at 0, 1, 2, 3, 6, 9 days.

It can be seen that after MJ treatment, the expression of related genes in SMB and SCT was up-regulated within a certain range. The genes measured in SCT are all up-regulated to varying degrees. Specifically, when MJ was treated for 6, 9 days, the expression levels of *TAT*, *RAS*, and *C4H* were significantly higher than those of the control group. When MJ was treated for 9 days, the expression of *HPPR*, *CYP98A14*, *4CL* was significantly higher than that of the control group, and when MJ was treated for 3 days, the expression of *4CL* was significantly higher than that of the control group. The expression level of *PAL* was significantly higher than that of the control group after 1 day of MJ treatment. The response of related genes in SMB to MJ is weaker than that of SCT. When MJ was treated for 2 days, the expression of *TAT* and *HPPR* was significantly higher than that of the control group. In addition, when MJ was treated for 3 and 9 days, the expression of *TAT* was also significantly higher than that of the control group. However, when MJ was treated for 2 days, the expression of *PAL* was significantly higher than that of the control group, and *4CL* also showed the same changing trend when MJ was treated for 9 days.

## Discussion

### ^1^H-NMR determination of phenolic acids in SMB and SCT

Consistent with previous reports, the content of rosmarinic acid in SCT is higher than that in SMB (Cheng et al. 2005). After MJ treatment, the content of related phenolic acids in SMB and SCT increased. That is different from the previous report (Fang et al. 2017), we found that MJ can promote the accumulation of rosmarinic acid and related phenolic acids in SMB within a certain treatment time. From an overall comparison, the phenolic acids in SCT have a higher response to MJ than SMB. The promotion effect is the most significant when MJ is treated for 9 days. To better compare the differences in the mechanism of action of MJ on SMB and SCT, the qRT-PCR technology was used to analyze the expression of several key genes.

### Gene expression was analyzed by qRT-PCR

After MJ treatment, the expression of related genes in SMB and SCT both showed different degrees of up-regulation within a certain processing time. In addition to the previously reported *TAT*, *HPPR*, *RAS* three genes (Fang et al. 2017), we also found that the expression of *C4H*, *4CL* and *CYP98A14* in SCT were up-regulated, but the SMB changes are different. However, the up-regulation of *PAL* gene expression previously reported (Li et al. 2012) is not very clear in our experimental results. On day 1 of MJ treatment, the expression of *PAL* in SCT was significantly up-regulated, but on days 2 and 3, the expression of *PAL* in SMB and SCT was significantly down-regulated, respectively.

MJ significantly up-regulated the expression of *4CL*, *TAT*, *HPPR*, *RAS*, *C4H*, *PAL*, *CYP98A14* in SCT within a certain treatment time, but only significantly up-regulated the expression of *TAT* and *HPPR* in SMB, and significantly down-regulated the expression levels of *PAL* and *4CL*. That is to say, MJ plays a role in both the phenylalanine and tyrosine branches of the rosmarinic acid synthesis pathway, and promotes the accumulation of rosmarinic acid by up-regulating the expression of related genes in the two branches. MJ mainly acts on the tyrosine branch in the process of rosmarinic acid synthesis in SMB, and promotes the accumulation of rosmarinic acid by up-regulating the expression of related genes. To a certain extent, this seems to provide exploration ideas for the differential response of SMB and SCT to MJ.

**Fig. 8:**
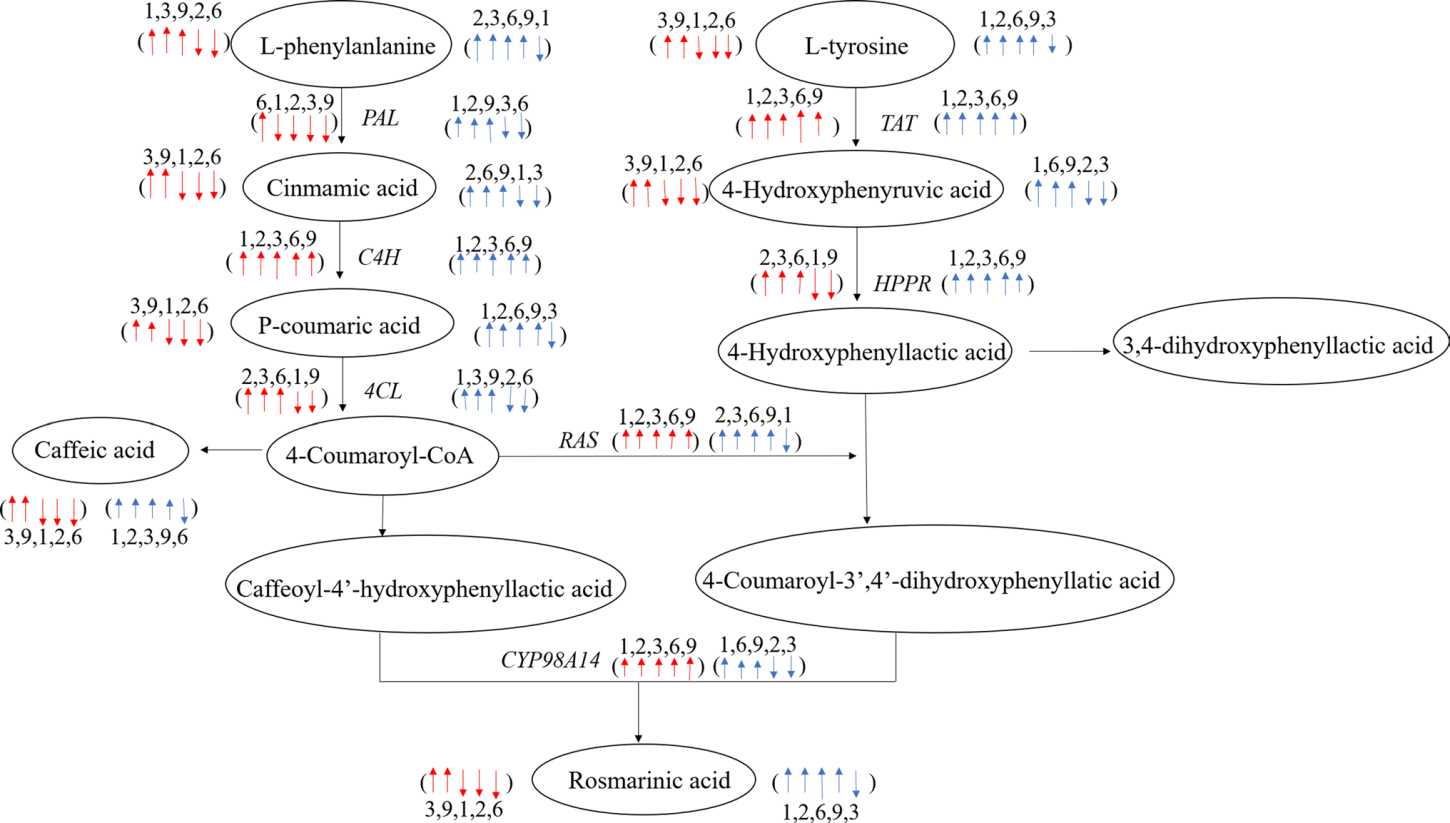
The effect of MJ on the synthesis of SMB and SCT rosmarinic acid. Among them, the red arrow represents SMB, and the blue arrow represents SCT. Up and down respectively represent the changes in the corresponding components of the MJ treatment group compared with the control group, and the number above the arrow corresponds to the number of days of MJ treatment

To more intuitively compare the difference between SMB and SCT on MJ, we summarized the experimental results shown in Fig. 8. It can be seen that whether it is SMB or SCT, after MJ treatment, related substrates and gene expression in the rosmarinic acid synthesis pathway were increased to varying degrees. For SMB, the contents of l-phenylalanine, cinnamic acid, *p*-coumaric acid and caffeic acid on the phenylalanine branch and l-tyrosine, 4-hydroxyphenylpyruvic acid on the tyrosine branch and rosmarinic acid were higher compared with the control group on the 3 and 9 days of MJ treatment. Related gene expression did not show a consistent trend. It is more obvious that the expression levels of *C4H*, *TAT RAS* and *CYP98A14* were higher than those of the control group during the entire time range of MJ treatment. Compared with SMB, SCT had a stronger response to MJ. Specifically, within 5 days of MJ treatment, the contents of l-phenylalanine, *p*-coumaric acid, caffeic acid, l-tyrosine and rosmarinic acid were higher than the control group for 4 days, the contents of cinnamic acid and 4-hydroxyphenylpyruvic acid were also higher than the control group for 3 days. Intriguingly, the expression level of *HPPR* in SCT also continued to be higher than that of the control group, but *RAS* and *CYP98A14* did not show a tendency to always be higher than the control group in SCT. In summary, MJ has a significant promoting effect on the metabolism of rosmarinic acid in SMB and SCT, and its effect on SCT is greater than SMB. Based on this, we made the following conjectures: the differential response of secondary metabolism-related genes to the induction of MJ may lead to different salvianolic acid metabolic responses between the two.

## Concluding remarks

To explore the mechanism of MJ on rosmarinic acid biosynthesis in SMB and SCT, we used ^1^H-NMR technology and qRT-PCR technology to obtain the content of phenolic acids in the hairy root of SMB and SCT under different treatment times with MJ, and combined with the expression of related genes for analysis. Compared with the control group, we found that MJ can promote both of the synthesis of rosmarinic acid and related components in its synthesis pathway in SMB and SCT. We found that MJ significantly increased the expression of *TAT* and *HPPR* in the synthesis pathway of rosmarinic acid in SMB, and significantly increased the expression of *TAT*, *RAS*, *C4H*, *PAL*, *4CL*, *CYP98A14* and *HPPR* in SCT. Compared with SMB, SCT has a stronger response to MJ. That is to say, MJ promotes the expression of related genes in the phenylalanine and tyrosine branches of rosmarinic acid synthesis in SCT, but only up-regulates the expression of *TAT* and *HPPR* genes in the tyrosine branch of SMB. This is probably due to the different reactions induced by the secondary metabolism-related genes to methyl jasmonate leading to the different metabolic reactions of salvianolic acid between the two. This discovery not only provides new ideas for the mechanism of action of MJ on rosmarinic acid, but also provides important ideas for the synthesis of other phenolic acids. Of course, there are some meaningful issues that are worthy of in-depth exploration in this research. For example, why is the expression of *PAL* and *4CL* on the phenylalanine branch significantly down-regulated after MJ induction? Will there be changes in the response of related genes to MJ at 0–24 h increasing the sampling time point and are the changes the same? We believe that these questions can be a focus of the next research, and the analysis of this problem will provide more ideas and basis for the biosynthesis of rosmarinic acid and even phenolic acids by MJ.

### *Author contribution statement*

Formal analysis, YL; funding acquisition, ZH and LX; project administration, ZL; resources, FS; software, JC; supervision, XZ and DY; writing—original draft, YL; writing—review and editing, ZH. All authors have read and agreed to the published version of the manuscript.

## Electronic supplementary material

Below is the link to the electronic supplementary material.

Supplementary material 1 (DOCX 642 kb)

## Abbreviations

C4H: Cinnamate 4-hydroxylase
4CL: 4-Coumarate: CoA ligase
CYP98A14: Cytochrome P450
HPPR: 4-Hydroxyphenylpyruvate reductase
MJ: Methyl jasmonate
PAL: Phenylalanine ammonia-lyase
PAS: Rosmarinic acid synthase
SCT: *Salvia castanea* f. *tomentosa* Stib
SMB: *Salvia miltiorrhiza* Bunge
TAT: Tyrosine aminotransferase
