# Supplementary material for: Application of 1H-NMR combined with qRT-PCR technology in the exploration of rosmarinic acid biosynthesis in hair roots of Salvia miltiorrhiza Bunge and Salvia castanea f. tomentosa Stib
Source: Planta. 2020 Nov 27;253(1):2. doi: 10.1007/s00425-020-03506-y (PMC7695671; doi:10.1007/s00425-020-03506-y)
Supplement: Supplementary file 1 — Supplementary material 1 (DOCX 642 kb) [file 425_2020_3506_MOESM1_ESM.docx]

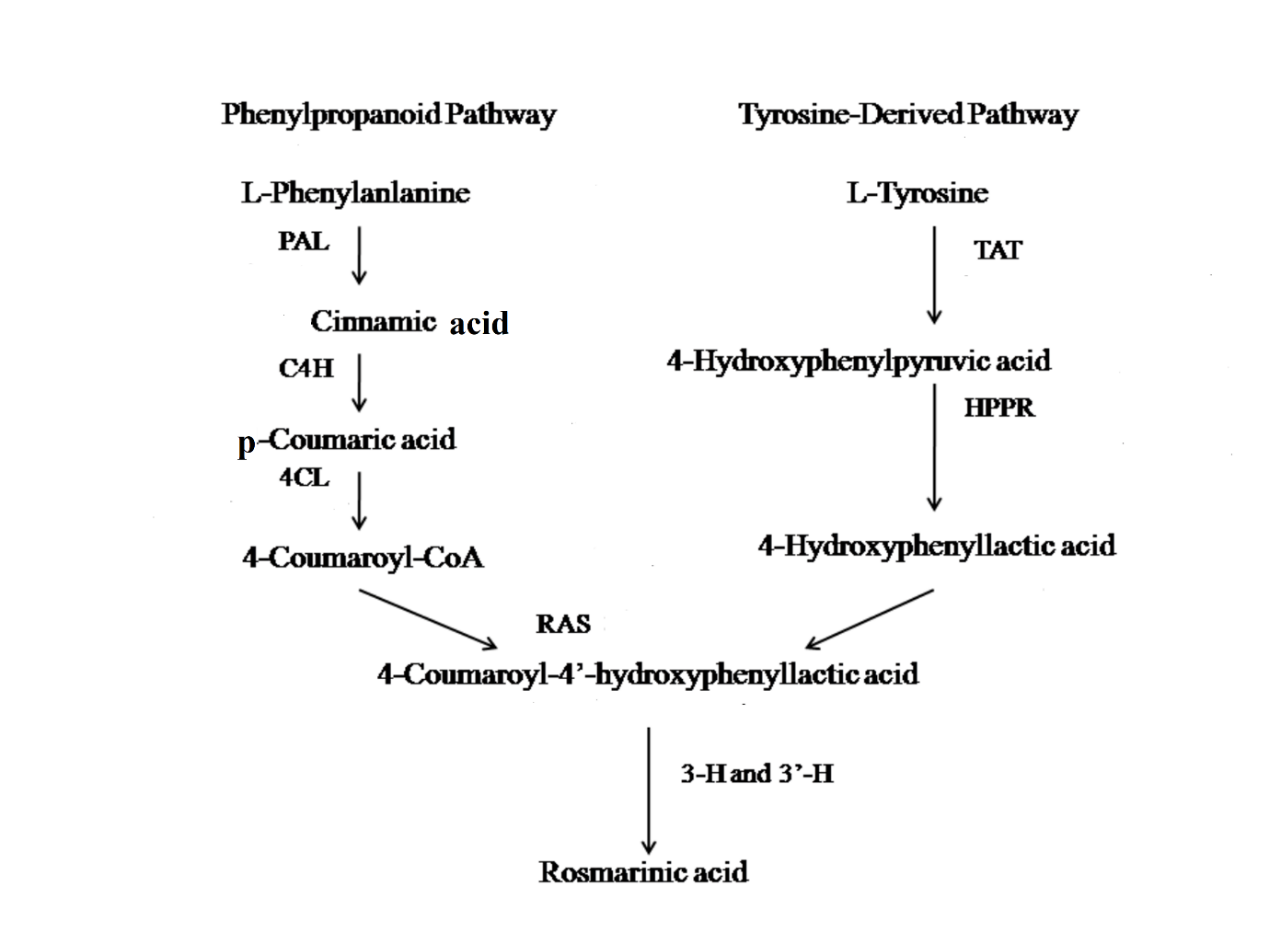


**Fig. S1** Rosmarinic acid biosynthetic pathway.


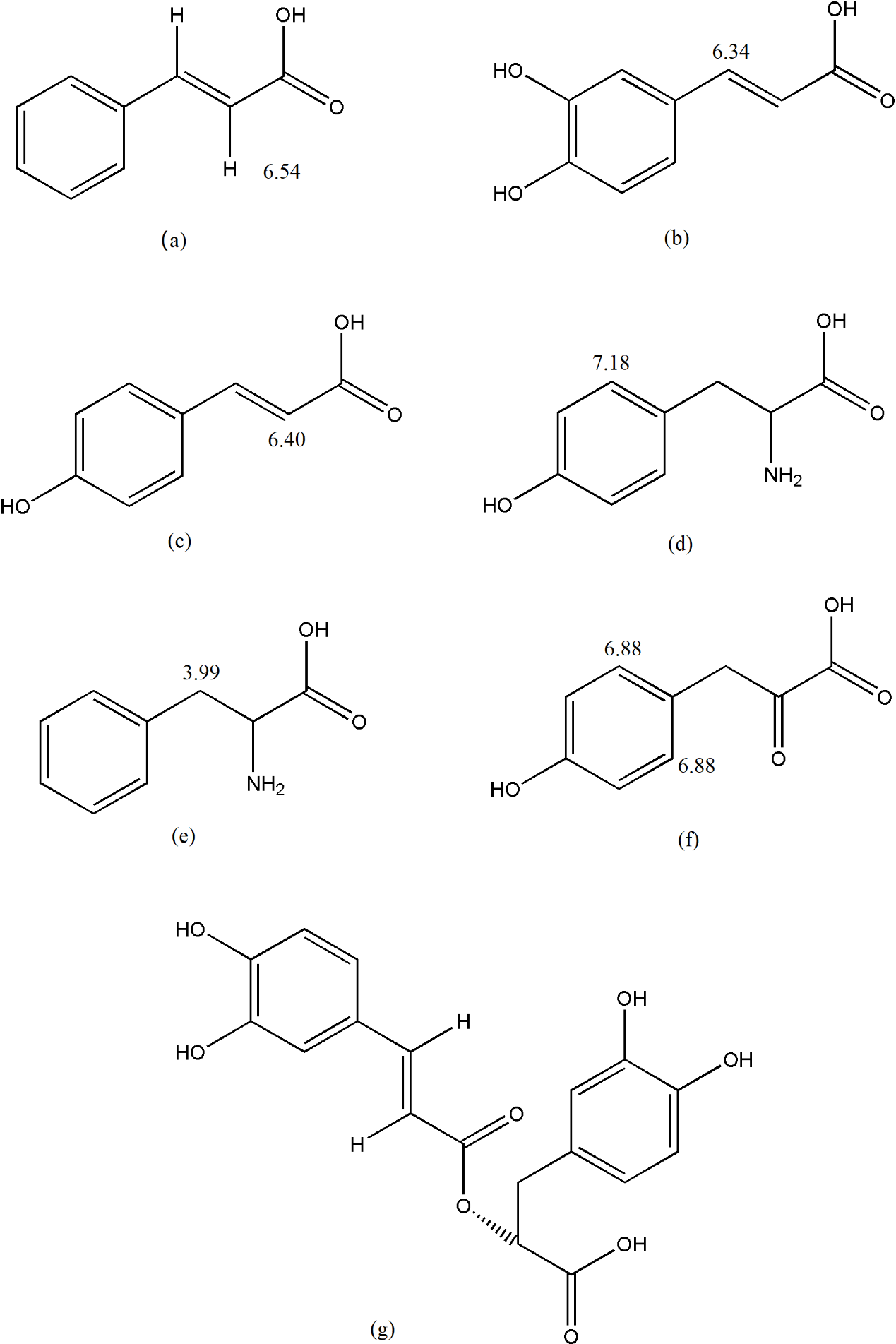


**Fig. S2** Chemical structures of cinnamic acid (**a**), caffeic acid (**b**), p-coumaric acid (**c**), L-tyrosine (**d**), L-phenylalanine (**e**), 4-hydroxyphenylpyruvic acid (**f**) and rosmarinic acid (**g**). (The numbers in the figure represent the chemical shifts of the marked peaks of the NMR spectrum of each compound, unit is ppm.)
